# Influenza virus DI particles: Defective interfering or delightfully interesting?

**DOI:** 10.1371/journal.ppat.1008436

**Published:** 2020-05-21

**Authors:** Fadi G. Alnaji, Christopher B. Brooke

**Affiliations:** 1 Department of Microbiology, University of Illinois at Urbana-Champaign, Urbana, Illinois, United States of America; 2 Carl R. Woese Institute for Genomic Biology, University of Illinois at Urbana-Champaign, Urbana, Illinois, United States of America; University of Wisconsin Madison, UNITED STATES

Influenza virus (IV) defective interfering particles (DIPs) were first described by Preben Von Magnus in the 1940s as a noninfectious form of the virus that accumulated during infection [1,2]. Subsequent studies revealed that most, if not all, virus families are capable of producing defective particles with properties similar to IV DIPs [3]. Despite decades of investigation, how—and more importantly, why—DIPs are produced during IV infection remains a mystery.

## What is in a name?

IV DIPs are characterized by large internal deletions within 1 or more genome segments that render them incapable of independent replication (hence the “defective” descriptor). The “interfering” comes from the well-characterized ability of DIPs to directly inhibit the replication of wild-type (WT) virus by outcompeting WT gene segments for replication resources and packaging. These phenotypic classifications have become more complicated over time. Replication-incompetent particles are not necessarily “defective” from the standpoint of a virus population as they can contribute to viral replication through collective interactions [4,5]. It is also not clear that all IV particles with deletions have interfering activity. For simplicity’s sake, we will use the term DelVG (deletion-containing viral genome) to refer to any viral genome segment containing a large (>10 nucleotides) deletion and DIP to refer to any particle harboring a DelVG.

## How are DelVGs made?

DelVGs and DIPs occur naturally during virus infection and can be easily generated and propagated in the lab. There are 2 separate processes involved in DIP generation: (1) the formation of DelVGs during genome replication and (2) the packaging and propagation of DelVGs within DIPs. For the sake of brevity, we will only discuss the formation of DelVGs here.

Influenza DelVGs are likely formed by viral polymerase errors that occur during the replication process, rather than by ligation of viral RNA fragments or splicing [3,6]. This is supported by 3 main findings: first, canonical splice donor/acceptor sequences are not observed at DelVG deletion junctions (the point at which the remaining sequences flanking the deletion connect to each other)[7]. Second, DIP abundance can be significantly affected by specific amino acid substitutions in the viral polymerase [8,9]. Third, the 5′ and 3′ sequences of each DelVG are always derived from the same segment and polarity [10], ruling out formation through random ligation. A recent study showed that the elimination of foreign genes from the viral genome occurs via deletion within a single molecule rather than recombination between 2 viral template molecules [11], suggesting that DelVGs are also derived from a single template molecule.

**Fig 1:**
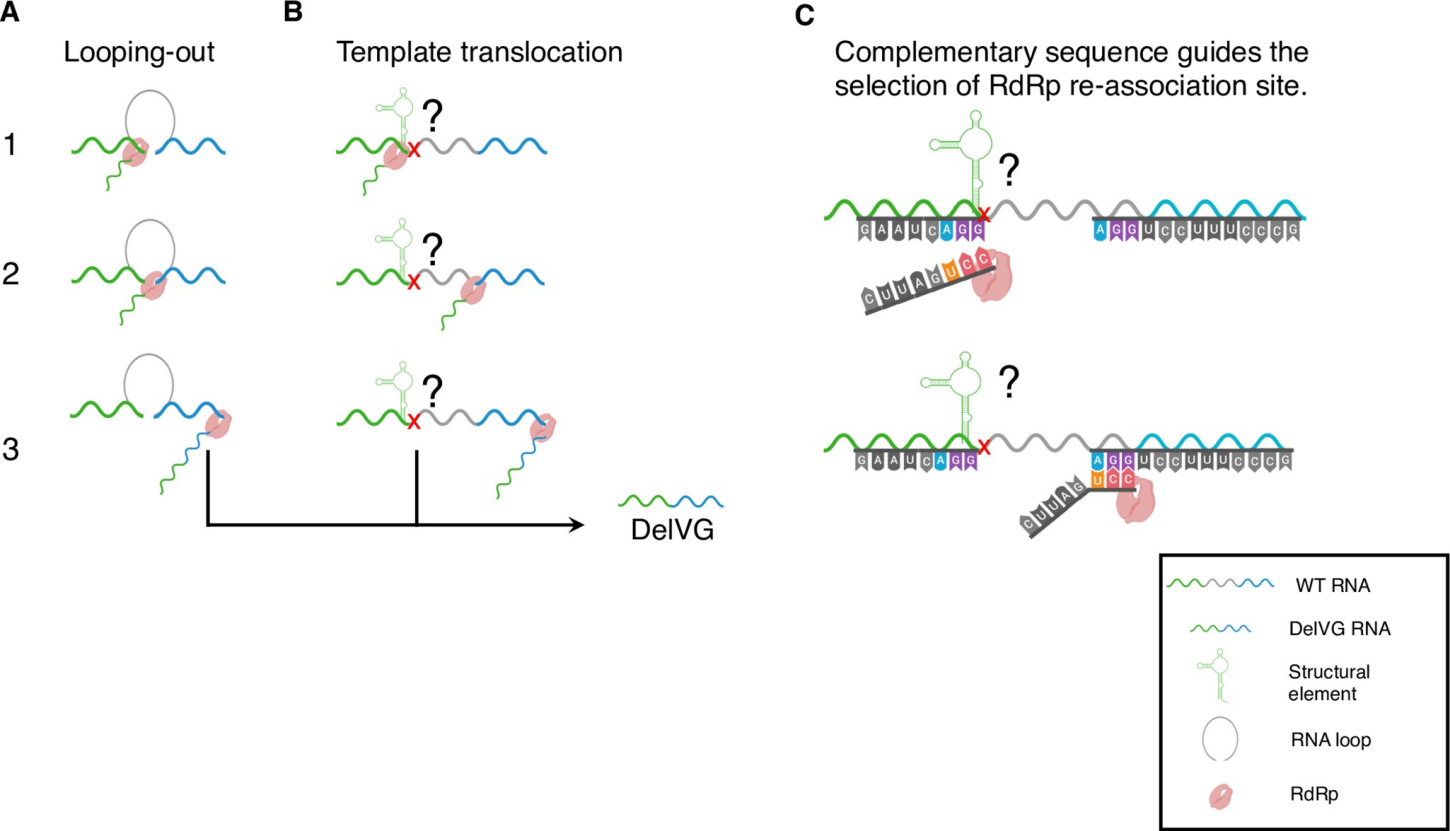
Proposed DelVG formation mechanisms for influenza viruses. **(A) Looping-out model**. The 2 sites of the DelVG junction are brought together by an RNA loop structure (1), potentially facilitated by the hairpin structure of the viral RNP, creating a connection point where the viral polymerase complex can “roll-over” (2) resulting in deleting the loop sequence (3). **(B) Template translocation model**. Pausing of daughter strand synthesis by the RdRp (1) is suggested to be potentially caused by a local RNA structure in the template or basepair mismatch at the junction site (the red x). RdRP remains associated with nascent daughter strand and continues processing along the template molecule (2). At a downstream point, the RdRp resumes templated elongation of the daughter strand (3), resulting in deletion. **(C)** A schematic based on model B showing how complementarity within overlapping sequences at the DelVG junction sites may allow the nascent strand to re-associate with the template, where the RdRp can resume elongation. *Figures generated with the assistance of Biorender*.*com*. DelVG, deletion-containing viral genome; RdRp, RNA-dependent-RNA-polymerase; WT, wild type

Two main models have been proposed for how this process might work at the molecular level. The first model, called “looping-out”, suggested that an RNA structure could bring 2 distant sites of a gene segment strand close to each other, forming a loop that the polymerase can skip without dissociating from the template or the nascent strand (Fig 1A) [6]. However, recent advances in our understanding of the influenza RNA-dependent-RNA-polymerase (RdRp) complex structure have revealed that template RNAs must pass through fully enclosed channels to access the active site [12–14], preventing the easy association/dissociation of polymerase and template that is possible with some other viral polymerase structures. These structural constraints on template mobility are hard to reconcile with the “looping-out” model and instead support an alternative model in which DelVGs form when the RdRp pauses synthesis of the nascent daughter strand while continuing to process along the template molecule and then resumes daughter strand synthesis at a downstream point on the template (Fig 1B) [7,15].

The specific molecular determinants that trigger the pausing of polymerization remain unclear. Potential candidates include the incorporation of base mismatches or local RNA structure. The downstream reinitiation of polymerization has been proposed to be guided by complementarity between the nascent strand and the site of reinitiation on the template (Fig 1C). Support for this model comes from the recent demonstration that such complementarity is important for the formation of mini viral RNAs (mvRNA; another aberrant viral RNA product with much larger deletions than canonical DelVGs) [16]. mvRNAs differ from DelVGs in that they are much shorter and can be replicated in the absence of viral nucleoprotein, and it is not clear how much mechanistic overlap exists in the formation of mvRNAs and DelVGs.

Analysis of DelVG sequences has provided contradictory information about the importance of complementarity at DelVG deletion junctions. Two recent studies reported the presence of overlapping sequences at the flanking regions of IV DelVG junction sites [17,18]. In contrast, we and others failed to detect a significant enrichment of overlapping sequences at the junction sites of many DelVGs [19,20]. We compared hundreds of actual DelVG junctions generated during infection with a simulated dataset of randomized junctions and found no significant difference in the occurrence of complementarity between the 2 samples.

In summary, the specific molecular determinants that govern the formation of DelVG remain a mystery. There are also several outstanding questions about the patterns of DelVG formation across the genome. For instance, we and others have shown that DelVGs form much more readily from some genome segments compared with others and that these patterns can differ between viral strains and types [19]. One thing to keep in mind is that most of our knowledge of DelVGs comes from sequencing what has been packaged into DIPs. The need for DelVGs to get successfully packaged and propagated before we see them may be biasing our understanding of what actually gets produced by the viral polymerase. It is tempting to speculate that analyzing intracellular DelVGs isolated prior to packaging might reveal different patterns than those isolated from DIPs.

## Why are DIPs so common?

Beyond the mysteries of how DIPs form, the key question of why they form remains unresolved. For decades, the dogma was that DIPs, as indicated by their name, are defective products that interfere with WT virus replication through competition for resources and packaging [3]. They are also believed to potently activate host innate immunity, another feature that should be bad for overall viral fitness [3]. These features suggest that DIP production should be highly harmful for the virus.

In the past, this was explained by the idea that DIP production was simply an artifact of high multiplicity of infection (MOI; refers to the ratio of infecting virions to target cells) in vitro replication conditions, with minimal relevance for natural infection. Sustained high MOI infection creates conditions in which the emergence of DelVGs within viral populations might be inevitable [21]. Recent studies (examining both IV and paramyxoviruses) have upended this view by revealing that DIPs are actually a common product of human infection that may play an important role in influencing pathogenicity [9,17,22–24].

If DIP production is as harmful to the virus as believed, then there should be a strong selective pressure to minimize DIP production. Based on this assumption, we would expect that viruses that produce fewer DIPs to outcompete and numerically dominate those with greater DIP production. This raises the question of why DIPs appear to be so common during natural infection.

There are multiple possible explanations for why DIP production has not been eliminated by selection. For instance, DelVG production may be an unavoidable side-product of the influenza virus RNA replication process. The influenza virus polymerase may be unable to rapidly synthesize genomic RNAs without making occasional mistakes (i.e., deletions). A similar trade-off between replication speed and mutation rate has been proposed for poliovirus [25]. Arguing against this is the observation that some viral genome segments can apparently replicate just fine without producing appreciable DelVGs [19]. Alternatively, DIP production may not be as bad for the virus as long thought. As far as we know, no one has ever been able to directly test the effects of variation in DIP production on IV population fitness, and there is still a lot we do not understand about collective interactions within IV populations. In line with this, recent work demonstrating that DIPs can promote paramyxovirus persistence has clearly established how DIP production may benefit viruses in some circumstances [26].

After several decades of study, DIPs remain a weird and fascinating complication in our understanding of influenza biology, and numerous basic questions remain unanswered. First and foremost, how do DelVGs form, and what are the viral genetic determinants that govern their formation? Second, is DIP production as bad for the virus as has long been believed? How does the presence of DIPs alter the fitness and transmissibility of viral populations? Finally, how does DIP production influence that pathogenic potential of IV populations? The answers to these questions will greatly improve our fundamental understanding of influenza biology and are likely to reveal that DIPs are way more delightfully interesting than is commonly believed.
